# Social media, self-harm and suicide

**DOI:** 10.1192/bjb.2019.94

**Published:** 2020-08

**Authors:** Allan House

**Affiliations:** Leeds Institute of Health Sciences, School of Medicine, University of Leeds, UK

**Keywords:** Self-harm, suicide, social media, service users, information technologies

## Abstract

Use of social media by people with mental health problems, and especially those who are prone to self-harm, has potential advantages and disadvantages. This poses a dilemma about how and by how much the form and content of social media sites should be regulated. Unfortunately, participation in the public debate about this dilemma has been restricted and high-profile discussion of necessary action has been focused almost entirely on how much suppression of content is justified. Professional bodies, including the Royal College of Psychiatrists, should be doing much more than they are to shape how the debate is conducted.

## The dilemma

There are, broadly speaking, positive and negative accounts of how people with mental health problems, and especially those with a history of self-harm, experience social media.^1,2^ Negative accounts have been emphasised in mainstream media,^3^ with social media presented as places where you are drawn into an immersive atmosphere of depressive messages and images that act as enticement to self-harm and suicide. More positive accounts from young people suggest that social media can also offer a space where you can come out of hiding, share otherwise secret fears with peers, and gain an element of support and advice.^4^

In the former view, risk of suicide is increased by the mood-lowering effect of the content and by a sort of creeping familiarity with the idea of self-harm or suicide – called sometimes desensitisation or normalisation – and greater awareness of the methods involved. In the latter view, the social or networking function creates opportunity for reducing the sense of disconnection or lack of belonging, and the sharing of detail allows some alleviation of the burdensomeness of feeling uniquely troubled (these and other ideas about risk of suicide are discussed by Joiner^5^). This Janus-faced nature of social media is well outlined in a report by Barnardo's about young people, social media and mental health – *Left to Their Own Devices.*^6^

The natural conclusion is that different people are likely to be affected differently by their online experiences, and the same person may be affected differently on different occasions. Which raises the question – how to minimise risk without at the same time suppressing useful content? This is a dilemma that requires a careful public debate involving as many interested parties as possible in coming to a solution that considers all the competing demands of the situation.

There are three issues that contribute to the complexity of the problem.

First, examination of online material about self-harm reveals substantial diversity in form and content.^7^ Those who post and those who respond to posts are engaged in conversations not just about the manifest topic of self-harm and suicide, even when the relevant posts are explicitly tagged as self-harm: content is also about emotional problems more generally, about relationships, fitting in or belonging, and about attractiveness, sexuality and body image. The mixture of textual and visual messaging leads to communication the ambiguity and irony of which can be missed by reading one without the other.

Second, much of this may be regarded as helpful by those who access it,^4^ even when the content includes direct communication about self-harm with images of self-injury. Such images can help an isolated person (anything up to a half of people who self-harm do not seek help for their problems^8^) feel less alone. The images may come with messages about self-care or harm minimisation. It is reasonable to conclude that content which some people find unhelpful is found helpful by others, and that whether particular content is found to be helpful or unhelpful by a particular individual depends on the immediate circumstances in which it is accessed.

Third, it is not clear what the putative pathway to harm is, following exposure to self-harm material online. Words such as graphic, explicit or glamorising are in themselves not tightly defined, but they imply that the underlying mechanism is an invitation to copy the behaviour. Linking this argument to suicidal behaviour is problematic – for example most online images of self-harm are of self-injury (cutting or burning) and yet these are extremely rare methods of suicide, especially in young people. If the putative pathway to suicide is not by copying then presumably it is by exposure leading to low mood and hopelessness – in which case it is not clear that images of self-injury are more problematic than other mood-influencing content.

These sources of ambiguity raise the serious possibility that clumsy, excessive or inconsistent intervention – in the name of reducing harmful exposure and (by implication) habituation or normalisation – may have the unintended damaging consequence of increasing the sense of disconnectedness and burdensomeness experienced by people with mental health problems who self-harm.

## The public debate

In the UK, this public debate has centred recently on the suicide of teenager Molly Russell, not least because her father has pressed forcibly the case for the damaging effect of social media and the need to suppress content that might (in his view, definitely does) encourage suicide. The BBC opened their coverage with an aggressive interview^9^ of Steve Hatch, the managing director of Facebook in Northern Europe, by Amol Rajan, who is the BBC's Media Editor rather than somebody with expertise in self-harm or suicide. Not long afterwards the government produced a White Paper – *Online Harms*^10^ – that bundled encouraging self-harm or suicide with incitement to terrorist activities, dissemination of child pornography, and drug dealing on the dark web. The main direction has not therefore been about self-harm and suicide prevention, but about steps to regulate the tech giants.

The response from the principal player in this case – Facebook/Instagram – has been dispiriting. After an attempt to use Nick Clegg as a front man, they announced earlier this year a ban on images of self-harm described as graphic or explicit^11^ – with no definition of either offered by way of clarification. Now Instagram has announced a ban on drawings or cartoons depicting self-harm.^12^ There is again lack of clarity about exactly what this means: in the cited article the specific example is of text linked to an innocuous drawing. What is happening, in the absence of serious discussion of the pros and cons, is a piecemeal suppression of visual content of social media postings.

## The missing element: clinical and academic leadership

Where is the commission-like meeting of organisations, clinicians, academics and people with personal experience, that should be leading the debate and informing the decisions? Neither the social media companies nor the government has shown an interest in organising such an activity. The mainstream media, as one might expect, just want a story to tell, sentimental or sensational if possible. Samaritans has an interest^13^ but progress is painfully slow.

What is striking in all this is the absence from the debate of the professional bodies – the Royal College of Psychiatrists, the British Psychological Society, the Royal College of Nursing, the Health and Care Professions Council. Although members of the Royal College of Psychiatrists may have an online presence, and although the College has issued (rather one-sided) advice to its members^14^ and further guidance about professional standards of online behaviour is forthcoming, the College has conspicuously not been organising the high profile, mature debate that is needed to replace what is going on now. The absence of professional bodies from that role represents a failure of leadership in an area of public health where they should be at the forefront of educating the general public about self-harm and suicide, and modelling how intelligent decisions should be made on complex and important topics. It is time our College took the lead, initiating the establishment of a joint working group with the key professional bodies, third-sector organisations and people with personal experience. The role of such a body is not to establish the practicalities of regulation or control of content, but to offer the best available advice about what such content should be. The group should have a strong policy related to communication and dissemination of its discussions and not just await the production of a report – the aim is as much to model how discussion should happen as it is to achieve any other outcome.

## About the author

**Allan House** is Professor of Liaison Psychiatry at the Leeds Institute of Health Sciences, School of Medicine, University of Leeds, UK. He is author of *Understanding and Responding to Self-Harm: The One Stop Guide* (Profile Books, 2019), which offers practical advice for anyone affected by self-harm.
